# *Chlamydia pneumoniae* can infect the central nervous system via the olfactory and trigeminal nerves and contributes to Alzheimer’s disease risk

**DOI:** 10.1038/s41598-022-06749-9

**Published:** 2022-02-17

**Authors:** Anu Chacko, Ali Delbaz, Heidi Walkden, Souptik Basu, Charles W. Armitage, Tanja Eindorf, Logan K. Trim, Edith Miller, Nicholas P. West, James A. St John, Kenneth W. Beagley, Jenny A. K. Ekberg

**Affiliations:** 1 Menzies Health Institute Queensland, Griffith University, Gold Coast campus, Southport, QLD Australia; 2 School of Immunology and Microbial Sciences, King’s College London, London, UK; 3 Centre for Immunology and Infection Control, School of Biomedical Sciences, Queensland University of Technology, Brisbane, Australia; 4 Griffith Institute for Drug Discovery, Griffith University, Nathan campus, Brisbane Queensland, Australia

**Keywords:** Microbiology, Neuroscience

## Abstract

*Chlamydia pneumoniae* is a respiratory tract pathogen but can also infect the central nervous system (CNS). Recently, the link between *C. pneumoniae* CNS infection and late-onset dementia has become increasingly evident. In mice, CNS infection has been shown to occur weeks to months after intranasal inoculation. By isolating live *C. pneumoniae* from tissues and using immunohistochemistry, we show that *C. pneumoniae* can infect the olfactory and trigeminal nerves, olfactory bulb and brain within 72 h in mice. *C. pneumoniae* infection also resulted in dysregulation of key pathways involved in Alzheimer’s disease pathogenesis at 7 and 28 days after inoculation. Interestingly, amyloid beta accumulations were also detected adjacent to the *C. pneumoniae* inclusions in the olfactory system. Furthermore, injury to the nasal epithelium resulted in increased peripheral nerve and olfactory bulb infection, but did not alter general CNS infection. In vitro,* C. pneumoniae* was able to infect peripheral nerve and CNS glia. In summary, the nerves extending between the nasal cavity and the brain constitute invasion paths by which *C. pneumoniae* can rapidly invade the CNS likely by surviving in glia and leading to Aβ deposition.

## Introduction

*Chlamydia pneumoniae* is a gram-negative respiratory pathogen, responsible for causing 5–20% of community-acquired pneumonia^1,2^. *C. pneumoniae* primarily infects the pulmonary and nasal mucosa, but has in recent years been linked to diseases distinct from the respiratory tract, such as atherosclerosis/coronary disease^3^, asthma^4^, inflammatory arthritis^5^, multiple sclerosis^6^ and, in particular, late-onset dementia (late-onset Alzheimer’s disease)^7,8^.

Several key studies have reported the presence of *C. pneumoniae* in post-mortem brains from late-onset dementia patients. In the late 1990s, it was shown that 90% of such post-mortem patient brains contained *C. pneumoniae* DNA, compared with only 5% of control age-matched brains^9,10^. More recently, *C. pneumoniae* DNA was found in 80% of patient brains, compared to 11% of control brains^11^. The presence of *C. pneumoniae* in brains from patients with late-onset dementia has also been shown using immunohistochemistry, where *C. pneumoniae* antigens were found in proximity to classical hallmarks of late-onset dementia pathology; senile plaques, amyloid beta (Aβ) deposits and cells containing neurofibrillary tangles in the cerebral cortex and hippocampus. It is now well known that Aβ is an antimicrobial peptide, released by neural cells in response to infectious agents^12,13^, so it is not surprising that the presence of bacteria in the brain can result in Aβ deposition reviewed in^8^. Viable *C. pneumoniae* bacteria have also been isolated from some post-mortem patient brains^7^. In contrast, however, other studies have failed to detect higher levels of *C. pneumoniae* in post-mortem brains from patients with late-onset dementia^14^, although the analytical methods have varied between studies^7^. Studies in mice have also suggested a link between *C. pneumoniae* and late-onset dementia. *C. pneumoniae* DNA, antigen and/or live bacteria have been detected in the brain of inoculated mice^15–18^, which resulted in Aβ deposition^16,17^ or altered appearance of Aβ deposits^18^. Importantly, the mice used in these studies were wild-type mice and not mouse models of Alzheimer’s disease, suggesting that *C. pneumoniae* can contribute to the neuropathologies associated with late-onset dementia.

Regardless of whether *C. pneumoniae* is a contributing factor to neurodegeneration, it is clear that this bacterium can infect the brain and potentially contributes to chronic CNS pathologies. To date, it remains unknown exactly how *C. pneumoniae* reaches the CNS. *C. pneumoniae* can infect lung macrophages, which migrate through the mucosal barrier and enter the blood; the bacteria can disseminate to vasculature by surviving intracellularly in blood monocytes, which can then cross the blood–brain barrier (BBB)^19^. It is also, however, possible that *C. pneumoniae* may enter the CNS via alternative routes. The nerves that extend between the nasal cavity and the brain, the olfactory and trigeminal nerves, have been shown to be a path for CNS infection by some infectious agents reviewed in^20^. These two nerves connect with the brain at the olfactory bulb and the brainstem, respectively. Interestingly, the olfactory bulb, entorhinal cortex and hippocampal formation (all olfactory structures), as well as the brainstem, are the CNS regions that exhibit the earliest signs of pathology in both late-onset dementia and familial Alzheimer’s disease^21–23^.

After intranasal inoculation in mice, *C. pneumoniae* antigens and/or infectious organisms have been detected in the olfactory mucosa and the olfactory bulb 1–4 months post intranasal inoculation^16–18^, and *C. pneumoniae* DNA in these tissues has been detected 1 week after inoculation^18^, strongly suggesting that the bacteria can infect the CNS via the olfactory nerve. Certain other bacteria, however, have been shown to very rapidly (within days) reach the CNS via the olfactory and/or trigeminal nerves reviewed in^20^. We recently demonstrated that *Chlamydia muridarum* can quickly (within two days) enter the CNS via these paths^24^, suggesting that *C. pneumoniae* may also rapidly infect the brain via the nerves.

Furthermore, it is unknown how soon Aβ starts to accumulate after *C. pneumoniae* inoculation. Whilst previous studies have shown that this occurs after months^16–18^, if the bacteria reach the CNS more rapidly, alterations in Aβ deposition may also occur sooner. In a transgenic mouse model of familial Alzheimer’s disease (5xFAD mice, which exhibit the human amyloid precursor protein and presenilin 1 transgenes with five mutations linked to Alzheimer’s disease), intracranial injection with *Salmonella typhimurium* resulted in a dramatic increase in Aβ deposition after only 48 h^13^. Whilst Aβ deposition is of course much more pronounced in Alzheimer’s disease mouse models, Aβ secretion in response to pathogens by (wild-type) neural cells can be rapid^12^.

Even though cranial nerves constitute a direct path by which microbes can access the brain, CNS infections are relatively rare, and only a small number of infectious agents are thought capable of accessing the brain via these paths. The nerves are well-protected physically and immunologically by the nasal epithelium which exhibits powerful innate and adaptive immune system components. Together with the associated nasopharynx-associated lymphoid tissue (NALT), the epithelium constitutes the first defence against microbes^25^. Injuries to the nasal epithelium are, however, relatively common^26^ and may expose the underlying cranial nerves to infection. Experimental injuries to the nasal epithelium of mice has been shown to increase the risk of bacterial invasion of the olfactory nerve and bulb by some bacteria^27,28^. Most microbes are, however, likely eliminated by phagocytic glia, olfactory ensheathing cells (OECs) and trigeminal Schwann cells (TgSCs), should they penetrate the epithelium and reach the nerves^29–31^. The glia limitans layer between the peripheral nerves and brain, populated by astrocytes, constitutes a further immunological barrier against CNS infection^32,33^. Whilst it is largely unknown why certain infectious agents can infect the CNS via cranial nerves, one key mechanism is thought to be the ability of these pathogens to infect and survive in OECs, TgSCs and astrocytes, as well as in microglia (the main phagocytes inside CNS tissue)^20,27,28,34,35^.

*Chlamydiae* are obligate intracellular bacteria with a unique biphasic life-cycle reviewed in^36^. Outside of host cells, *Chlamydiae* exist as infectious, biologically inactive elementary bodies (EBs), which exhibit strong resistance to environmental stress. *C. pneumoniae* EBs can become internalized into host cells, including many phagocytes^1,37,38^. The EBs are resistant to endosomal/lysosomal degradation, and inside the host cell transform into reticulate bodies (RBs). RBs replicate in inclusions (modified cellular vacuoles), which expand in size as the bacteria replicate. After approximately 72 h (in cell culture), the RBs transform into EBs, which are released by cell lysis and can infect new cells (exit via extrusion of membrane-bound compartments can also occur^39^). *Chlamydiae* can also persistently infect cells^40^ which is likely relevant for the link to chronic diseases^1^. Persistent *Chlamydia* infection can last for many years, and the persistent *Chlamydia* bacteria can re-activate^41,42^.

In the current study, we investigated whether *C. pneumoniae* could rapidly (3–7 days after intranasal inoculation) invade the CNS via the olfactory and/or trigeminal nerves in mice and if this resulted in any alterations in Aβ deposition in nerve/CNS tissue. Furthermore, we investigated whether *C. pneumoniae* could infect and survive in cultured primary mouse OECs, TgSCs, astrocytes and microglia. We also determined whether *C. pneumoniae* infection had any role in the regulation of Alzheimer's disease gene expression over the longer term.

## Material and methods

### Bacterial strains

*Chlamydia pneumoniae* AR39 (ATCC 53592) is a human pharyngeal isolate and was propagated in Hep-2 cells (sourced from the ATCC CCL-23). *C. pneumoniae* stocks were harvested in sucrose phosphate glutamate **(**SPG) and aliquots were stored at – 80 °C for future experiments. The infectious yield of *C. pneumoniae* was determined by counting inclusion forming units (IFU) in HEp-2 cells.

### Animals

7–8 week old female BALB/c mice were sourced from Animal Resource Centre (ARC, Murdoch, Western Australia) and were intranasally inoculated (under anaesthesia with isofluorane 1.5–2%) with either 10 µL of PBS (phosphate buffer saline) as vehicle control (N = 5) or *C. pneumoniae* (1 × 10^6^ IFU [inclusion forming units] per mice, N = 12), delivered as a 5 µL droplet per nostril. Mice were then sacrificed 1, 3, 7 and 28 days post intranasal inoculation by asphyxiation with rising carbon dioxide.

### Nasal epithelium injury model

In this study, we also used the methimazole injury model to investigate whether epithelial injury can increase the risk of *C. pneumoniae* invasion of the olfactory/trigeminal nerves and brain. For this purpose, 7–8 week old female BALB/c mice were injected with a single dose of methimazole (50 mg/kg, 10 mg/mL in PBS) or vehicle (PBS only), according to our previously published protocol^27,29^. Three days after methimazole injection, animals were intranasally inoculated with *C. pneumoniae* (N = 12) or vehicle (N = 5) as outlined above.

### Tissue collection

Heads and tissues including the olfactory mucosa (containing the olfactory nerve fascicles), olfactory bulb, trigeminal nerve and the brain (the remainder of the brain after removal of olfactory bulbs) were collected from euthanized mice, 1, 3, 7 days and 28 days post inoculation, for bacterial load determination and histology.

### Organ load assay

**Figure 1:**
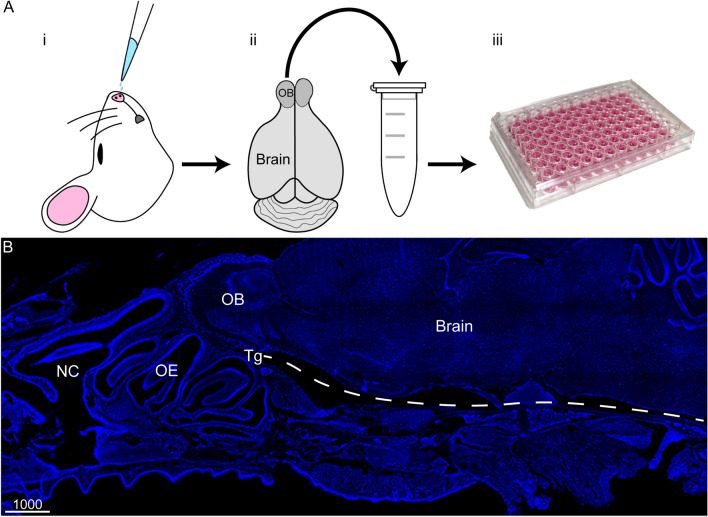
Schematics illustrating the process for quantifying the amount of viable infectious *C. pneumoniae* present in various mouse tissues. (**A**) Mice were first intranasally inoculated with *C. pneumoniae* (i), some with epithelial injury and some without. Following either 24 h, 3 days or 7 days or 28 days post inoculation, selected tissues were collected and homogenised in tubes (ii). Homogenates were serially diluted onto HEp-2 cells and incubated (iii). Cells were fixed and immunolabelled for *C. pneumoniae* inclusions, which were counted and the number of inclusion-forming units (IFUs) per mL of homogenate was determined. Data were then compiled into organ (tissue) load graphs (see Fig. 2). (**B**) Microscopic image showing a sagittal tissue section of a mouse brain. Cell nuclei/DNA are shown in blue (DAPI stain). Key anatomical locations include the nasal cavity (NC), olfactory epithelium (OE), olfactory bulb (OB), trigeminal nerve (Tg; not visible so approximate location is shown by white dotted line) and the brain. Scale bar in µm.

*Chlamydia pneumoniae* IFUs were detected by direct inoculation of tissue homogenate onto HEp-2 cells which were seeded on 96-well plates with 4000 cells/well. After 72 h, the *C. pneumoniae* inclusions in the entire wells were visualized by confocal microscopy and the numbers of IFUs isolated from the homogenates (IFU/mL) were determined (see workflow in Fig. 1).

### Chlamydial PCR

DNA was extracted from whole blood 2, 3 and 4 days after infection using the Qiagen DNeasy blood and tissue kit according the manufacturer’s instructions. The quantitative Real-time PCR was carried out by using Platinum SYBR Green qPCR SuperMix-UDG (ThemoFischer cat# 11733038). *C. pneumoniae* was detected using 16S rRNA primers (Forward: 5′-CTCAACCCCAAGTCAGCATT-3′and Reverse: 5′-CTACGCATTTCACCGCTACA-3′. The cycling program was 10 min at 95 °C, followed by 40 cycles of 15 s at 95 °C and 1 min at 60 °C, and a final dissociation stage. *C. pneumoniae* DNA was used as a positive control, and a no-template control was also included.

### Tissue preparation and sectioning

Heads were fixed in 4% paraformaldehyde (PFA) in PBS overnight at 4 °C, followed by decalcification in 20% ethylenediaminetetraacetic acid (EDTA) for 4 weeks. The heads were then embedded in optimal cutting temperature (OCT) medium and frozen. Sagittal sections (30 µm) were cut using a cryostat (Leica CM1860).

### Immunohistochemistry

Immunohistochemistry was performed as previously described^27,43,44^. Specimens were incubated with goat anti-*C. trachomatis/C. pneumoniae* (this antibody is used to detect both of these *Chlamydia* species; Abcam ab20929; 1:400) and/or rabbit anti-Aβ peptide (Abcam ab201060,1:500). Secondary antibodies were donkey anti-goat Alexa Fluor 488 (Abcam ab150129 1:400), donkey anti-rabbit 647 (Invitrogen A31573; 1:500). Antibodies were diluted in blocking buffer (2% bovine serum albumin with 0.3% Triton X-100 in PBS). Cryostat sections were first incubated with blocking buffer for 60 min at room temperature, followed by overnight incubation with primary antibodies at 4 °C. Sections were washed for 3 × 5 min, then incubated with secondary antibodies for 1 h. Cell nuclei were stained with 4′6-diamidino-2-pheylindole (DAPI).

### Primary glia cell culture

Olfactory ensheathing cells (OECs), trigeminal Schwann cells (TgSCs), astrocytes and microglia were used in this study. OECs and TgSCs were prepared from postnatal day 7 (P7) S100ß-DsRed transgenic mice; we have previously generated and described this transgenic mouse line and the cell isolation method^45^. Astrocytes and microglia were prepared from postnatal day 3 (P3) S100ß-DsRed transgenic mice following a previous published protocol^46^. S100ß-DsRed transgenic reporter mice were used due to the expression of DsRed fluorescent protein which is driven by the human S100ß promoter, such that glial cells including OECs, Schwann cells, astrocytes and microglia express DsRed protein and facilitate easy visualisation and identification in culture. The entire brain cell population was isolated from the brain tissue by enzymatic digestion and mechanical dissociation using Neural Tissue Dissociation Kit with GentleMACS (Miltenyi Biotec, 130-093-231). The cell pellet consisting of a mixture of all brain cells was further subjected to magnetic cells sorting for microglia enrichment using CD11b/c microbeads (Miltenyi Biotec, 130-093-636) or for astrocytes using anti-GLAST (ACSA-1) microbeads kit (Miltenyi Biotec, 130–095-825) according to manufacturer’s protocol. The different glial preparations were separately plated in plastic 24-well plates and maintained in glial medium containing Dulbecco's Modified Eagle Medium with 10% foetal bovine serum (FBS), G5 supplement (Gibco), gentamycin (Gibco, 50 mg/mL) and l-glutamine (200 μM) at 37 °C with 5% CO_2_ for 5 days. Cells were replated into T-25 flasks and allowed to proliferate to ~ 80% confluency. Primary glial cultures with approximately 70–80% purity was used in the experiments.

### In vitro infection of primary glial cells

Dilutions of *C. pneumoniae* bacteria were prepared in Dulbecco’s phosphate buffered saline (DPBS). OECs, TgSCs, astrocytes and microglia were seeded at the density of 4000 cells/well in 96-well plate (Costar) in glial medium. After 24 h, bacteria (multiplicity of infection (MOI): 1:1) were added and incubated with cells for 72 h. Following the infection, the cells were then rinsed in 1 × DPBS and were fixed for 20 min in 4% PFA in DPBS. Subsequently, cells were washed and incubated in blocking buffer for 1 h. Cells were then incubated with the following primary antibodies overnight at 4 °C; goat anti-*C. pneumoniae/Chlamydia trachomatis* (Abcam, ab20929; 1:400) and rabbit anti-glial fibrillary acidic protein (GFAP) antibody (Thermofisher Scientific, PA5-16291; 1:200) or rabbit anti-ionized calcium-binding adaptor molecule 1 (IBA1) microglia (Abcam, ab178847; 1:100). The following day, cells were washed with DPBS and incubated with secondary antibody donkey anti-goat Alexa Fluor 488 (Thermofisher Scientific, A11055; 1:400) and goat anti-rabbit 647 (ThermoFisher Scientific, A32733; 1:400) for 1 h. Nuclei were stained with DAPI. Hep-2 cells were visualized by CellMask Orange Plasma membrane stain (Thermofisher Scientific, C100455; 1:10,000).

### Viability assay for glial cells

Primary glial cells were infected with *C. pneumoniae* as described above. Cultures were harvested after 72 h in SPG with 5 mM l-glutamine and stored at − 80 °C. Culture plates were thawed and probe sonicated for 10 s (Sonics Vibra-Cell VCX 130, amp 1). Cell lysates (containing bacteria) were collected and serially diluted on a monolayer of HEp-2 cells and, 72 h later, washed with PBS and fixed with 4% PFA. Following immunocytochemistry, the infectious yield *C. pneumoniae* was determined by counting of the inclusion forming units (IFU) ml^−1^ in Hep-2 cells.

### RNA extraction and nanostring nCounter gene expression analysis

7–8 week old female BALB/c mice were infected as previously described. Mice were then sacrificed at 7 and 28 days post intranasal inoculation by asphyxiation with rising carbon dioxide. RNA from brain lysate (the remainder of the brain after removal of olfactory bulbs) of 7 and 28 days post *C. pneumoniae* inoculation and control mice (N = 3 for all groups) was extracted using Maxwell^®^ RSC simplyRNA tissue kits (Promega, AS1340) using the manufacturer’s protocol. RNA was eluted in 50 µL of nuclease-free water and quality/quantity of RNA was assessed. Following RNA elution, gene expression analysis was undertaken using the NanoString nCounter analysis system (NanoString Technologies, Seattle, WA) using the commercially available nCounter Alzheimer’s disease panel kit (Cat number: XT-CSO-MAD1-12). The Alzheimer’s disease panel contains 23 neurodegeneration pathways, targeting 770 genes including 10 internal reference/housekeeping genes. A master mix was made following manufacturer’s protocol with 70ul hybridisation buffer added to Reporter probes. Individual reactions for each sample were made with 8 μL master mix, 5 μL extracted RNA diluted to 125 ng and 2 μL Capture probe. Each reaction was hybridised in a thermal cycler (Eppendorf) at 65 °C for 20 h. Samples were processed on the NanoString Prep Station and the target-probe complex was immobilised onto the analysis cartridge. Cartridges were scanned by the nCounter Digital Analyser for digital counting of molecular barcodes corresponding to each target at 555 fields of view.

### Image capture

Images were captured using Nikon confocal microscope and Olympus FV3000 laser scanning confocal microscope. Three-dimensional reconstructions were made using Imaris × 64 (Version 7.4.2). For comparison between groups, the same image capture settings, laser intensity and focal depths were used. Images were colour balanced uniformly across the field of view using Adobe Photoshop Creative Cloud 2019 (20.0.4) and compiled into panels using Adobe Illustrator Creative Cloud 2019 (23.0.3).

### Statistical analysis

Data are shown as means ± SEM. Statistical significance was analyzed using either a two-way ANOVA with Bonferroni’s post hoc test or a one-way ANOVA with Tukey’s post hoc test. Statistical analysis was performed using GraphPad Prism 9.0 software, and statistical significance was set at *p* < 0.05.

Gene expression data was processed using the Advanced Analysis Module in the nSolver Analysis Software version 4.0 from NanoString Technologies (NanoString Technologies, WA, USA). Quality control was assessed, and the data was analysed using Rosalind software (partner open-source software). Normalised data were generated by the software followed by fold change and p value. p value was adjusted using Benjamini–Hochberg method of estimating false discovery rate. Venn diagram was generated using an open-source software (http://bioinformatics.psb.ugent.be/webtools/Venn) normalised to day 7 and day 28 non-infected control. Principal component analysis (PCA) and volcano plot was generated using Graphpad prism 9.0. Hierarchical clustering was generated using Morpheus software with Pearson correlation and average linkage method across the samples for the most significant genes (adjusted p value < 0.05). Molecular process was generated from REACTOME database linked to Rosalind software with cut-off set at p value < 0.05. Pathway profile score was generated from nSolver Analysis Software using the 23 neurodegenerative pathways mentioned previously.

### Ethics and biosafety

The experimental procedures used in the study were conducted with the approval of the Griffith University Biosafety Committee (NLRD/09/15_var7) and the Griffith University Animal Ethics Committee (MSC/08/18/AEC) in accordance with guidelines of the Australian Commonwealth Office of Gene Technology Regulator and the National Health and Medical Research Council of Australia. All the animal experiments in this study are reported in accordance with ARRIVE guidelines (https://arriveguidelines.org).

## Results

### *Chlamydia pneumoniae* infects the olfactory mucosa, olfactory bulb and cerebral cortex within 3 days after intranasal inoculation

The key aim of the current study was to determine whether *C. pneumoniae* could invade the brain via the olfactory/trigeminal nerve routes in the shorter term (≤ 1 week) after intranasal exposure, as has been shown for *C. muridarum*^24^. We intranasally inoculated adult mice with *C. pneumoniae*, then 3 days, 7 days and 28 days later mice were sacrificed. The 3 day time-point was chosen as *C. pneumoniae* has a life-cycle of approximately 72 h^1,37^. We then determined whether infectious (viable, inclusion-forming) *C. pneumoniae* could be isolated from the following homogenized mouse tissues: (1) olfactory mucosa (consisting of the neuroepithelium, underlying lamina propria and the many nerve fascicles that constitute the olfactory nerve), (2) olfactory bulb, (3) trigeminal nerve and (4) brain (beyond the olfactory bulb), as outlined in Fig. 1. Tissue homogenates were diluted onto HEp-2 cells and inclusion-forming units per mL of tissue homogenate were determined after 72 h.

**Figure 2:**
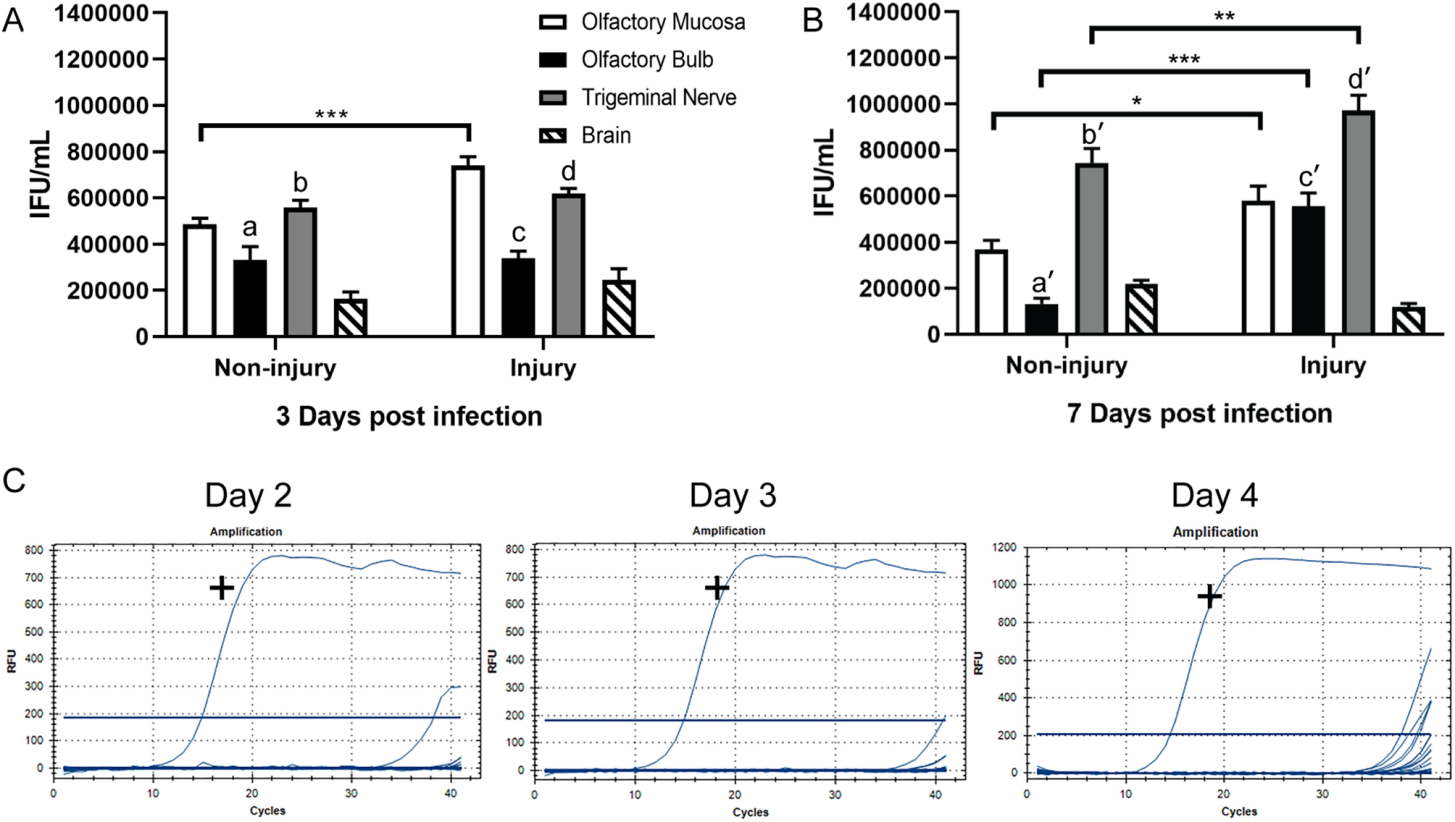
*C. pneumoniae* infects the nasal peripheral nerves and brain in mice with or without pre-injured nasal epithelium after intranasal inoculation. (**A**,**B**) Graph showing the amounts of *C. pneumoniae* IFUs isolated from various tissues of mice with or without pre-injured olfactory epithelium at 3 and 7 days post *C. pneumoniae* inoculation. Infectious *C. pneumoniae* organisms were isolated from the olfactory mucosa, olfactory bulb, trigeminal nerve and brain (n = 9/group). No *C. pneumoniae* was isolated from various tissues of control mice (vehicle only, n = 2). Data are shown as the mean number of inclusions ± SEM, n = 9/group. *p ≤ 0.05, **p ≤ 0.01 ***p ≤ 0.001, two-way ANOVA with Bonferroni’s post hoc test. For comparisons between non-injury Day 3 versus Day 7: a-a′ p ≤ 0.01, b-b′ p ≤ 0.01; for comparisons between injury Day 3 versus Day 7: c–c′ p ≤ 0.01, d-d′ p ≤ 0.001, two-way ANOVA with Bonferroni’s post hoc test. (**C**) PCR amplification curves of blood for *C. pneumoniae* (Cpn) at 2, 3 and 4 days post intranasal inoculation. (+) shows the positive control in each graph. Samples tested include the control (vehicle only; N = 2), methimazole only (N = 2), methimazole + Cpn (N = 9) and Cpn only (N = 9).

At 3 and 7 day time-points, infectious *C. pneumoniae* were isolated from all four tissues (Fig. 2A). No *C. pneumoniae* were detected in tissue lysate from control (vehicle-inoculated) mice (n = 2). For the olfactory bulb, the number of IFUs was significantly higher at 3 days than 7 days post inoculation (p.i.) (Fig. 2A,B), whereas the reverse occurred for the trigeminal nerve (Fig. 2A,B non-injury). At 28 days, low levels of infectious *C. pneumoniae* were isolated only from trigeminal nerve p.i. (not shown). We also determined whether *C. pneumoniae* was present in the blood 2, 3 and 4 days post intranasal inoculation using PCR. Based on the curves, all samples showed an absence of *C. pneumoniae* in blood (Fig. 2C). Note: the injury results presented in Fig. 2A,B are reported in the section “Injury to the nasal epithelium increases peripheral infection”.

**Figure 3:**
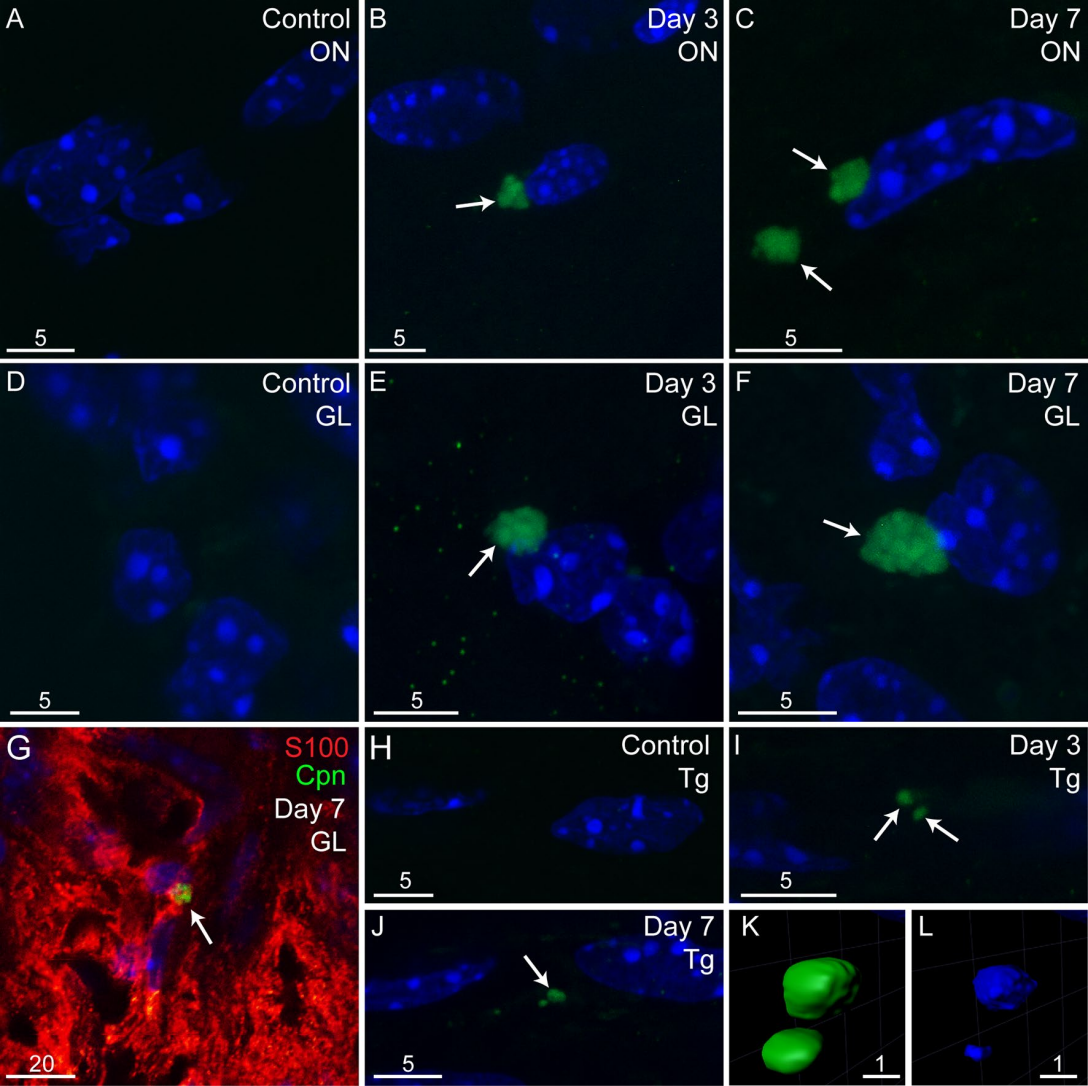
*C. pneumoniae* infects the nasal peripheral nerves and brain in mice after intranasal inoculation. Panels show images of tissue sections from control (vehicle only) and inoculated mice (*C. pneumoniae*) for both 3 days and 7 days post inoculation (p.i.). Images are representative ones from the olfactory nerve (ON) and olfactory bulb (OB) of 3 control mice, 3 *C. pneumoniae*-inoculated mice at 3 days p.i. and 3 inoculated mice at 7 days p.i. *C. pneumoniae* inclusions are shown in green (*C. pneumoniae* immunolabelling) and indicated by arrows. Nuclei/DNA is shown in blue (DAPI stain). Panels show maximum projection of confocal microscopy z-stacks. (**A**–**C**) Olfactory nerve (ON). (**A**) Control (vehicle only). (**B**) 3 days p.i. (**C**) 7 days p.i. (**D**–**F**) The glomerular layer (GL) of the OB. (**D**) control. (**E**) 3 days p.i. (**F**) 7 days p.i. (**G**) The GL at 7 days p.i., immunostained with anti-S100 antibodies. (**H**–**L**) Trigeminal nerve (Tg). (**H**) control. I: 3 days p.i. (**J**) 7 days p.i. (**K**,**L**) 3D reconstruction of panel J. *C. pneumoniae* inclusions. (**K**) Inclusions (green) are within the Tg and contain DNA (**L**; blue, DAPI stain). Scale bars in µm. For a low-power image showing the approximate localization of the images, see Fig. 1B.

We also analysed tissue sections from the olfactory nerve, olfactory bulb, trigeminal nerve and brain (beyond the olfactory bulb) for the presence of *C. pneumoniae* using immunohistochemistry. In addition to the 3 days, 7 days and 28 days mice, we also examined mice that had been sacrificed only 24 h after intranasal inoculation. 24 h after inoculation *C. pneumoniae* was detected within the olfactory mucosa and olfactory bulb and infectious *C. pneumoniae* were isolated from both the olfactory mucosa and olfactory bulb (Supplementary Fig. 1). However, as the bacteria at 24 h was likely to be from the inoculum we did not analyse this tissue further. At later time-points which are sufficient for at least one life cycle, *C. pneumoniae* inclusions were detected in the olfactory nerve (Fig. 3A–C), glomerular layer of olfactory bulb (Fig. 3D–F) and trigeminal nerve (Fig. 3H–L) at both 3 and 7 days p.i. Within the olfactory bulb, *C. pneumoniae* was only detected within the nerve fibre layer and glomerular layer, with *C. pneumoniae* inclusion bodies being present inside OECs (Fig. 3G). *C. pneumoniae* was also detected in the glomerular layer of the olfactory bulb at 28 days (Fig. 5Q).

Despite being able to isolate viable *C. pneumoniae* from the brain (beyond the bulb), we did not find definitive *C. pneumoniae* inclusions in brain tissue sections from these mice (not shown), suggesting that inclusions in brain tissue were too small or sparse to be confirmed by histology when screening tissue sections.

### Injury to the nasal epithelium increases peripheral infection

It has previously been shown that experimental injury to the olfactory neuroepithelium facilitates invasion of the olfactory nerve and bulb by certain bacteria^27,28^. To investigate whether epithelial injury could also affect *C. pneumoniae* infection of nerves and brain, we used our well-established methimazole-mediated model of nasal epithelial injury. Methimazole causes degeneration of the nasal epithelium in rodents^47^. We have shown that this mode of injury leads to patchy, dispersed injuries to the epithelium, separated by normal epithelium; this constitutes a model better resembling “natural” nasal injuries than other models, such as chemical irrigation models^27,29^.

**Figure 4:**
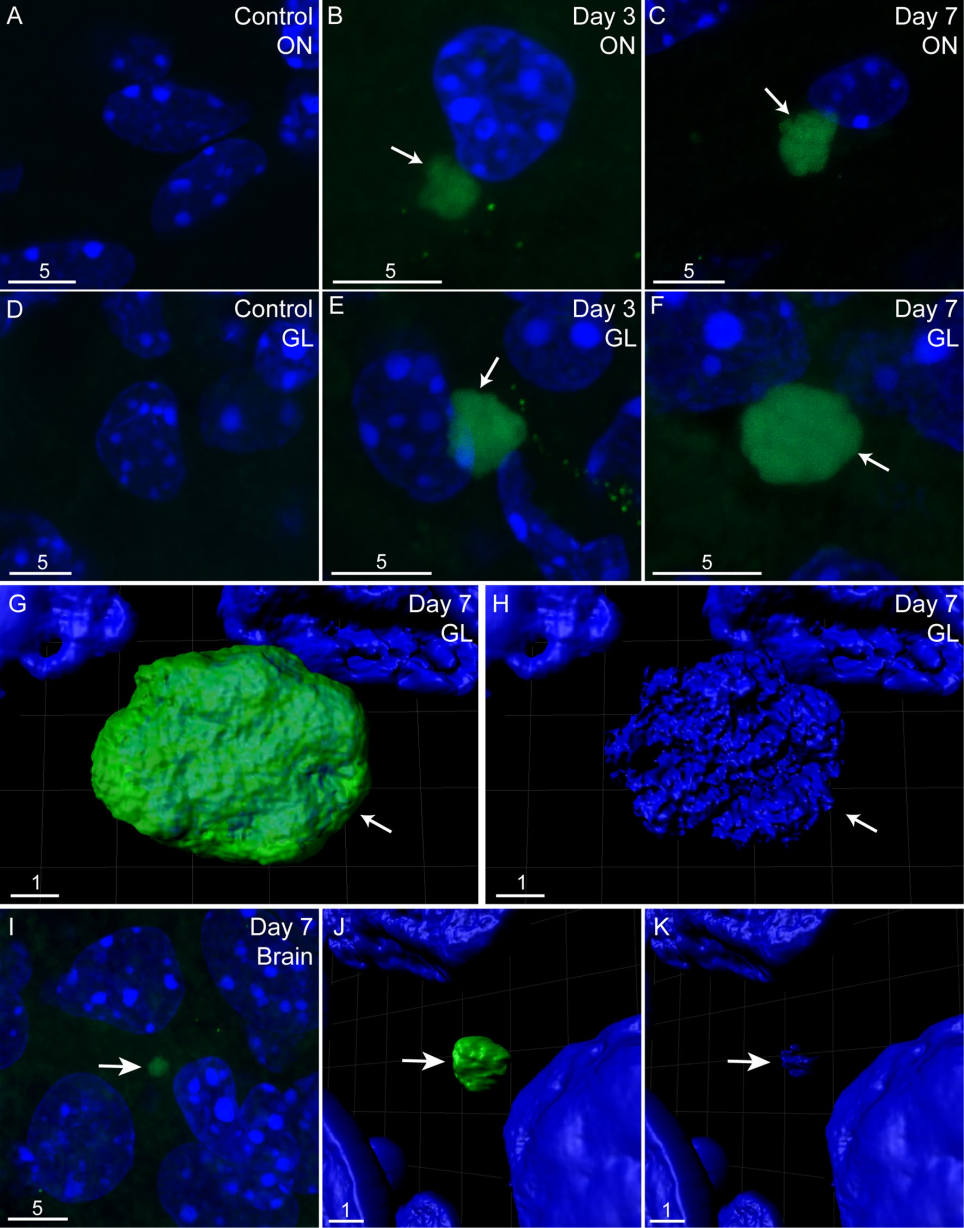
*C. pneumoniae* infection of the olfactory nerve, olfactory bulb and brain after injury to the olfactory epithelium. Panels show confocal images (maximum projection of z-stacks, **A**–**I**) of tissue sections from vehicle control and *C. pneumoniae*-inoculated mice, all with pre-injured olfactory epithelium before inoculation/vehicle treatment. Images are representative from n = 3 animals per group. *C. pneumoniae* inclusions are shown in green (immunolabelling) with nuclei/DNA in blue (DAPI stain). (**A**–**C**) The olfactory nerve (ON) in (**A**) control mice and mice inoculated with *C. pneumoniae,* (**B**) 3 days and (**C**) 7 days post inoculation. (**D**–**F**) The glomerular layer (GL) of the olfactory bulb in (**D**) control mice and inoculated mice (**E**) 3 days and (**F**) 7 days after inoculation. (**G**) A 3D reconstruction of the *C. pneumoniae* inclusion in panel (**F**) (green; arrow); (**H**) the same 3D reconstruction as panel (**G**), showing only the DAPI staining (blue), where bacterial DNA within *C. pneumoniae* inclusion is distinct from host cell DNA (arrow pointing to bacterial DNA). (**I**) Image showing a *C. pneumoniae* inclusion in the olfactory piriform cortex seven days after inoculation (green; arrow); (**J**,**K**) a 3D reconstruction and render of the *C. pneumoniae* inclusion shown in (**I**), with *C. pneumoniae* in green (**J**) and bacterial DNA shown in **K**, arrow). Scale bars in µm.

Mice were treated with methimazole, and 3 days later, when epithelial degeneration peaks^48^, the mice were inoculated intranasally with *C. pneumoniae*. The 3-day time-point was also chosen to limit any potential unrelated effects of methimazole, as methimazole at this stage has been largely cleared^49^. We examined mice that had been sacrificed only 24 h after intranasal inoculation. The methimazole treatment clearly damaged the epithelial layer (Fig. 4A–C) and *C. pneumoniae* was found within the lamina propria underlying the epithelial layer and in the nerve fibre layer of the olfactory bulb (Supplementary Fig. 2); as the bacteria is likely to be from the inoculum we did not analyse this tissue further.

Mice were then sacrificed 3 and 7 days after inoculation, followed by determination of the amounts of viable *C. pneumoniae* (IFUs) in tissues (Fig. 2A,B), as well as immunohistochemistry of tissue sections (Fig. 4). Whilst described separately here for better clarity, these experiments were conducted simultaneously to the experiment groups described for Fig. 2 (so that methimazole-induced epithelial injury followed by *C. pneumoniae* inoculation could be compared to *C. pneumoniae* inoculation alone).

We compared the *C. pneumoniae* load (IFUs) between (1) mice inoculated with *C. pneumoniae* alone and (2) mice with pre-injured nasal epithelium. Epithelial injury resulted in an increased *C. pneumoniae* load in the olfactory mucosa (which includes the olfactory nerve; both at day 3 and day 7 post inoculation), olfactory bulb and trigeminal nerve (day 7 only) in comparison to mice without injury (Fig. 2A,B). However, epithelial injury did not result in a significant difference in *C. pneumoniae* load in the brain.

For both the pre-injured mouse group and the group that was not pre-injured, the bacterial load in the trigeminal nerve was higher on day 7 than day 3 post inoculation (Fig. 2A,B). In the pre-injured group, olfactory bulb infection also increased with time, whereas the opposite occurred in mice that were not pre-injured (Fig. 2A,B). Immunolabelling confirmed the presence of *C. pneumoniae* in the olfactory nerve (Fig. 4A–C) and bulb (Fig. 4D–H). Three-dimensional reconstructions provided clear visualisation of the inclusions within the glomerular layer, with the DNA of the bacteria being clearly distinct from the host cell DNA (Fig. 4G,H). In one mouse, in the pre-injured group, *C. pneumoniae* IB/s were also found in the olfactory piriform cortex, with three-dimensional reconstruction again showing the bacterial DNA being distinct from the host cell DNA (Fig. 4I–K).

### Aβ is associated with regions of *C. pneumoniae* infection in the olfactory bulb

**Figure 5:**
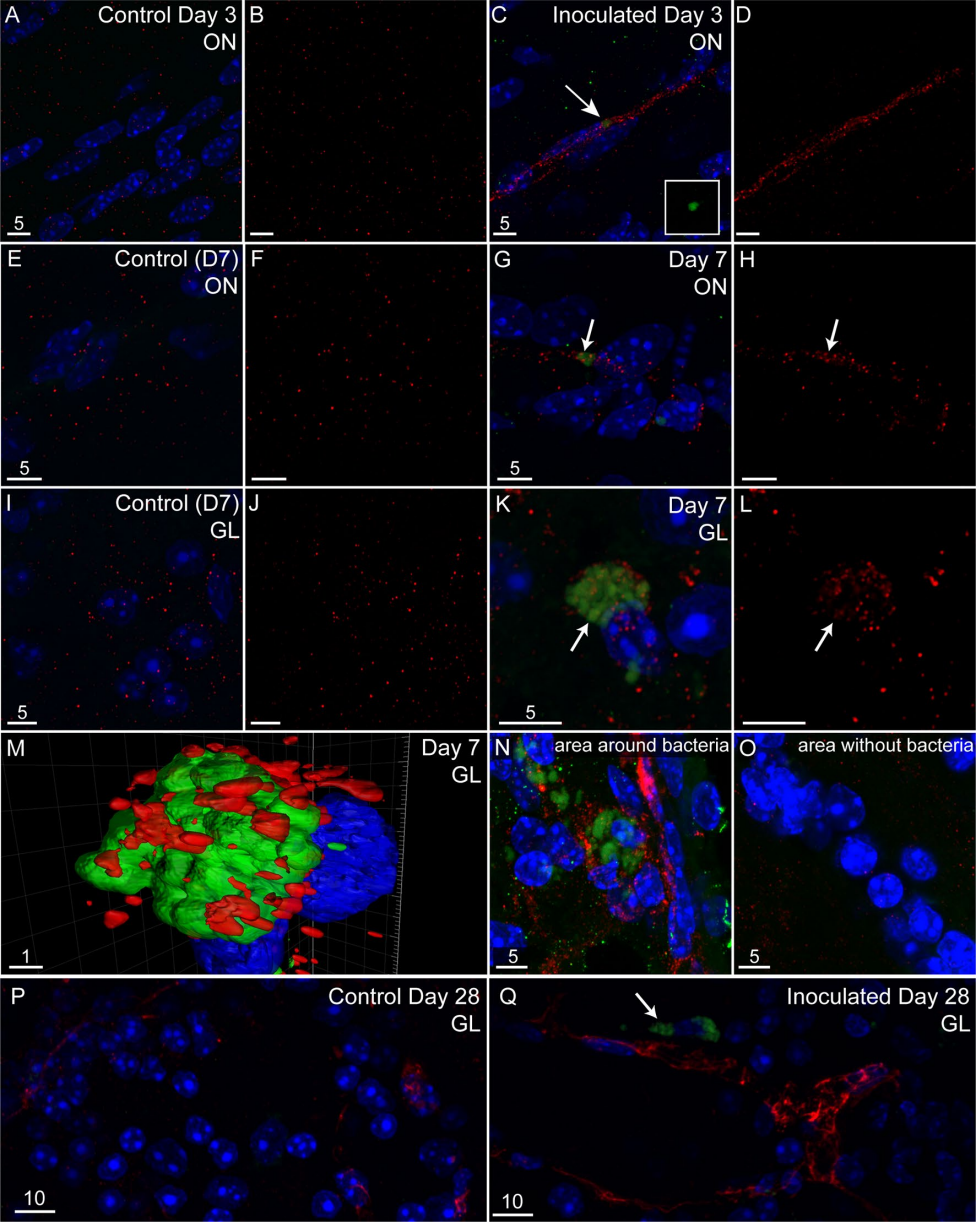
*C. pneumoniae* is associated with Aβ peptide accumulation in both the olfactory nerve (ON) and the glomerular layer (GL) of the olfactory bulb (OB). Images show maximum projections of confocal microscopy z-stacks from vehicle control mice and *C. pneumoniae*-inoculated mice, 3, 7 and 28 days p.i. Images are representative ones from the ON or OB of 3 control and 3 inoculated mice. Immunolabelling shows Aβ peptide (anti-Aβ1-42) (red), *C. pneumoniae* (green) and DNA (DAPI, blue). (**A**–**D**) At 3 days, the ON of (**A**,**B**) a control mouse and (**C**,**D**) a *C. pneumoniae*-inoculated mouse. Arrows show the location of *C. pneumoniae* inclusions (green in **C**). Panels (**B**) and (**D**) show only Aβ immunolabelling (red). (**E**–**H**) Similarly, at 7 days the immunolabelling of the ON in (**E**,**F**) control and (**E**,**F**) inoculated mice. (**I**–**L**) At 7 days, the GL in (**I**,**J**) a control mouse and (**K**,**L**) a *C. pneumoniae*-inoculated mouse. Panels (**J** and **L**): Aβ immunolabelling only (red). Arrows indicate *C. pneumoniae* inclusions. (**M**) A 3D reconstruction of panel (**K**) showing Aβ (red) surrounding a *C. pneumoniae* inclusion (green). (N–O) Images of GL regions within the same tissue section of a *C.* pneumoniae-inoculated mouse, showing areas where *C. pneumoniae* inclusions are, or are not, localized. (**N**) A GL area were *C. pneumoniae* inclusions were detected. Inclusions (green) were associated with Aβ accumulation (red). (**O**) An adjacent GL region where inclusions were not detected. Only diffuse Aβ immunolabelling is seen (red). (**P**,**Q**) At 28 days, the GL of (**P**) a control mouse and (**Q**) a *C. pneumoniae*-inoculated mouse. Arrows indicate *C. pneumoniae* inclusions; Aβ (red). Scale bars in µm.

To determine whether *C. pneumoniae* inclusions were associated with Aβ deposits, we also immunolabeled the sections for Aβ. We found diffuse/patchy Aβ immunolabelling in olfactory nerve and bulb tissues from all animals, including control mice (Fig. 5A,B,E,F,I,J,P). However, we found that in tissues from inoculated mice, distinct Aβ deposits accumulated near *C. pneumoniae* inclusions. At 3 and 7 days after inoculation, we detected Aβ deposits near *C. pneumoniae* inclusions in the olfactory nerve (Fig. 5C,D,G,H). At 7 days, Aβ deposits near *C. pneumoniae* inclusions were detected in the glomerular layer of the olfactory bulb (Fig. 5K–N). The Aβ deposits were not detected in adjacent tissue regions where inclusions were not present (Fig. 5O). At 28 days, Aβ deposits continued to be detected near *C. pneumoniae* inclusions in the glomerular layer of the olfactory bulb (Fig. 5Q), while control uninfected mice exhibited diffuse Aβ deposits (Fig. 5P) which is similar to previous reports^16,18,50^. *C. pneumoniae* inclusions, as well as associated Aβ deposits, were detected sporadically within tissues. For this reason, we were not able to quantify the difference in Aβ levels between tissues from the different time-points after inoculation, as well as between inoculated and control animals. Correlating with the fact that we could not detect *C. pneumoniae* inclusions in the brain beyond the olfactory bulb, we also did not detect any evidence of distinct Aβ deposits in these areas (not shown).

### *C. pneumoniae* can infect primary glial cells

**Figure 6:**
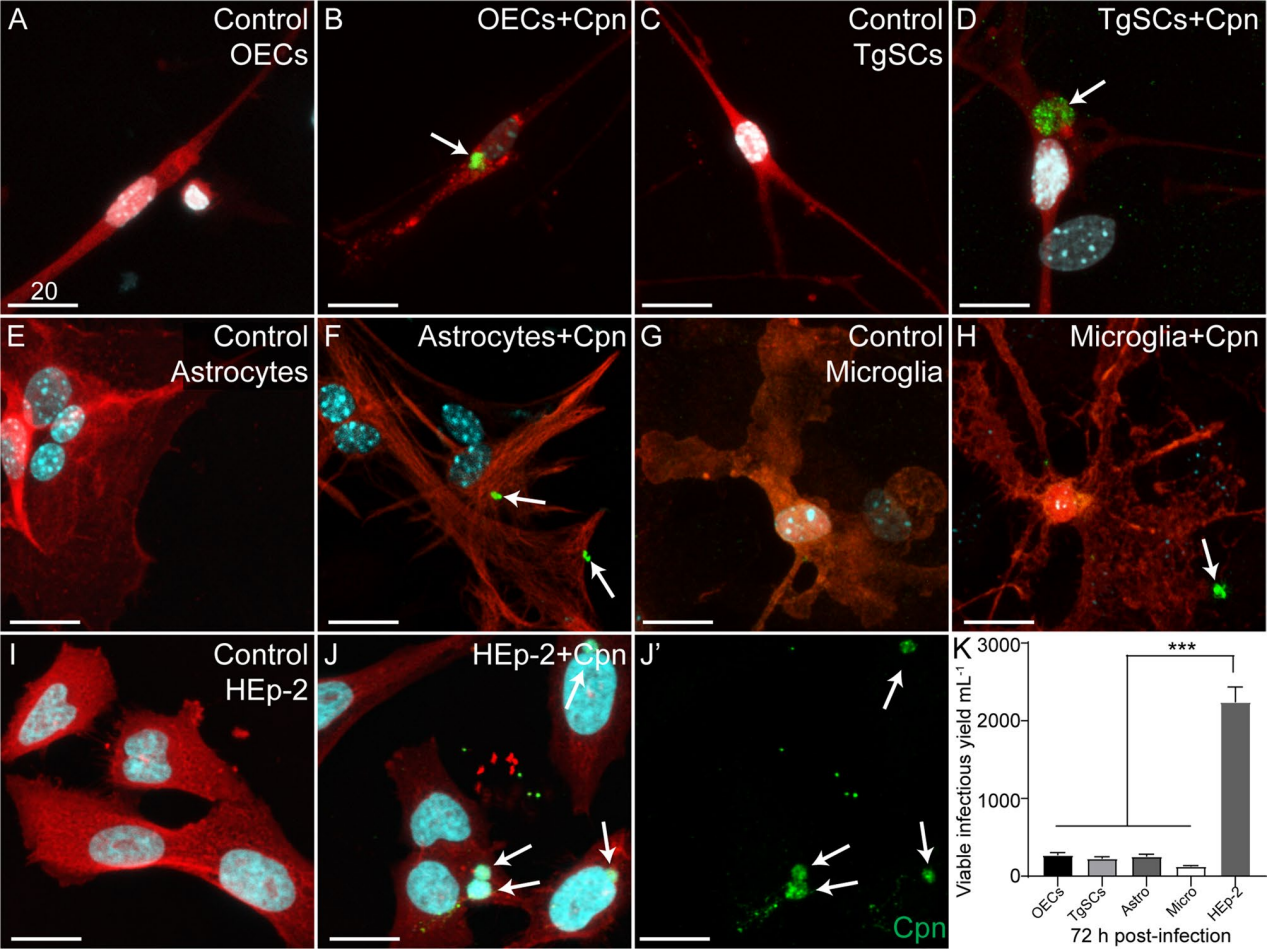
*C. pneumoniae* can infect glial cells from the olfactory and trigeminal nerves, olfactory bulb and brain. Cultured glia (OECs, TgSCs, astrocytes and microglia) from S100β-DsRed mice, in which glia express DsRed, were inoculated with *C. pneumoniae* (Cpn) for 72 h, along with HEp-2 cells. (**A**–**J**′) Maximum projection images from confocal microscopy z-stacks of control cells and *C. pneumoniae*-inoculated cells. Nuclei are stained with Hoechst (cyan). (**A**,**C**,**E**,**G**,**I**) Cells treated with cell culture medium alone (control; glia in red). (**B**,**D**,**F**,**H**,**J**) Cells inoculated with *C. pneumoniae*. Immunolabelling showed *C. pneumoniae* inclusions in the cells (green; arrows). (**J**-**J**′) Merged image shown in (**J**), *C. pneumoniae* inclusions alone shown in (**J′**). Scale bars in µm. (**K**) Graph showing the amounts of *C. pneumoniae* IFUs isolated from the cell cultures. The infectious yield of *C. pneumoniae* was significantly different between glia and HEp-2 cells (***p ≤ 0.001, one-way ANOVA with Tukey’s post hoc test). Data shown as the mean number of inclusions ± SEM.

The capacity to infect and survive inside glial cells is thought to be a key mechanism for the ability of bacteria to invade the CNS via cranial nerves^34,35^. Therefore, we next examined whether glia from the olfactory and trigeminal nerves, olfactory bulb and brain could constitute host cells for *C. pneumoniae* infection. OECs, TgSCs, astrocytes and microglia were inoculated with *C. pneumoniae* (MOI: 1:1, i.e., 1 IFU/cell) for 72 h. For comparison and as a positive control, HEp-2 cells, which are highly susceptible to *C. pneumoniae* infection and in which the bacteria have strong capacity for intracellular survival^51^, were also included. Cells were either (1) fixed and immunolabelled for *C. pneumoniae*, or (2) lysed for determination of *C. pneumoniae* IFUs (viable, infectious organisms). Immunolabelling showed *C. pneumoniae* inclusions in all cell types (Fig. 6A–J). The HE-p2 cells had distinctly more pronounced inclusions compared to the other cells, with the DNA of the bacteria being strongly visible (Fig. 6J-J’). The different glia had similar levels of viable *C. pneumoniae*, but significantly lower amounts of viable *C. pneumoniae* were recovered from all the glia than from HEp-2 cells (Fig. 6K).

### *C. pneumoniae* infection modulates Alzheimer’s disease related gene expression

**Figure 7:**
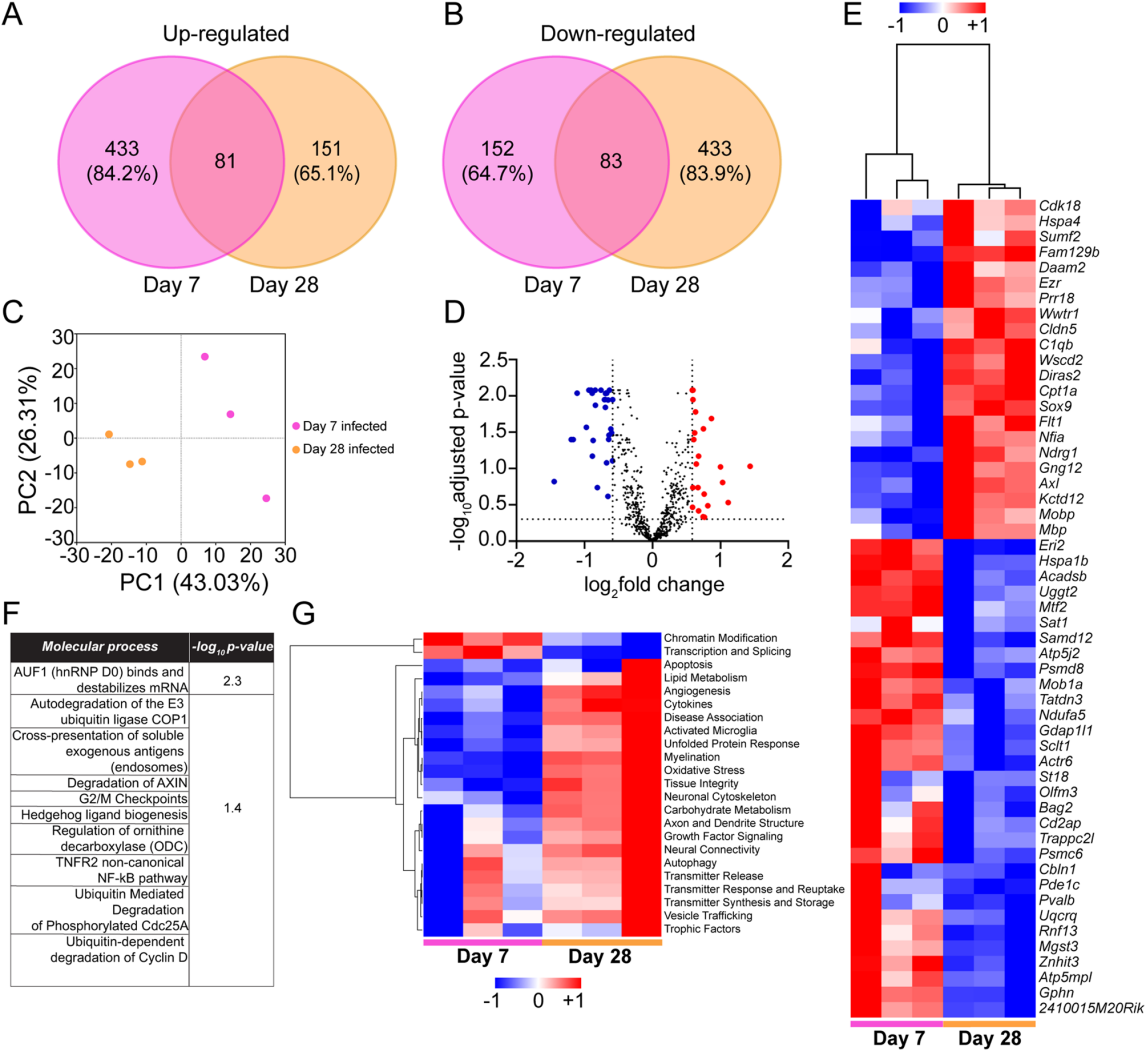
Differential gene expression (DGE) involved in Alzheimer’s disease for short (day 7) and long term (day 28) infection (n = 3/group). (**A**) Numbers and percentages of genes up-regulated for day 7 (pink) and day 28 (orange) infections normalised to their respective non-infected controls. (**B**) Numbers and percentages of downregulated genes for day 7 (pink) and day 28 (orange) infections normalised to their respective non-infected controls. (**C**) Principal component analysis of all the biological replicates of day 7 (pink) and day 28 infections (orange) for all gene expressions normalised to their respective controls. Axes show the percentage variation in each PC plots with maximum at 43.03% for PC1 followed by 26.31% for PC2. (**D**) Volcano plot of log twofold change of DGE of day 28 infection (normalised to day 7 infections) against –log 10 adjusted p value. Cut-offs for log twofold and –log 10 adjusted p value, set at ± 1.5 (± 0.585) and 0.5 (0.301), respectively. Blue dots represent down-regulated and red dots represent up-regulated genes. (**E**) Heat map of significant DGE plotted from (**D**) with hierarchical clustering on day 7 and day 28 infected replicates. Colour of the map is based on z-score from log twofold change for each gene (across rows). Blue represents down-regulated and red represents up-regulated genes. (**F**) Significant molecular processes (p < 0.05) in day 28 infection highlighted from REACTOME database with corresponding –log 10 p value. (**G**) Heat map of biological pathway profile scores compared between day 7 and day 28 infected samples from nSolver data for 23 pathways enlisted in Nanostring Alzheimer’s disease panel.

To investigate if *C. pneumoniae* infection had any role in the regulation of Alzheimer’s disease gene expression at the transcriptional level, we profiled 7 and day 28 day infected and non-infected mice brains using NanoString nCounter Alzheimer’s disease Panel. We first mapped the total number of genes which were up-regulated and down-regulated in day 7 and day 28 infected samples out of 760 genes which were all normalised to their respective non-infected samples. We found that a total of 514 genes were up-regulated in day 7 samples compared to 232 genes in day 28, out of which 81 genes were common to both and 433 (84.2%) were exclusively up-regulated in day 7 while 151 (65.1%) were exclusively up-regulated by day 28 (Fig. 7A). Interestingly, for genes that were down-regulated, 152 (64.7%) in day 7 and 433 (83.9%) genes in day 28 were exclusively down-regulated (Fig. 7B). We also investigated whether duration of infection had a role in differential gene expression (DGE) of Alzheimer’s disease genes. The normalised expression of all the 760 genes from day 7 and day 28 infected samples were used to construct correlation mapping using dimensional reduction technique (Principal component analysis—PCA). The maximum variance across the expressions was 43.03% (PC1 value) between the day 7 and day 28 infected samples (Fig. 7C). This analysis showed that the duration of infection had a definite role as the individual biological replicates from respective time points clustered together but away from each other as a group.

Next, we determined the significantly different genes with large changes in fold expression in infected samples of day 28 compared to day 7 in the form of a volcano plot. A volcano plot of the genes from day 28 infected samples using day 7 infected samples as a baseline and cut-offs at ± 1.5 on log twofold change and 0.5 on − log 10 adjusted p value was performed (Fig. 7D). A total of 53 genes were differentially regulated out of which 22 genes were down-regulated (blue) and 31 genes were up-regulated (red). We further explored these genes to investigate the relationship between the infected samples at the respective time points. A heatmap was constructed based on the z-score of the normalised gene expressions followed by hierarchical clustering using Pearson correlation method (Fig. 7E). We observed separate gene clustering of day 7 and day 28 infected mice. A total of 107 molecular processes were regulated (from REACTOME database using the Rosalind software) based on the DGE of day 28 infection compared to day 7. Out of these, 10 pathways were sorted based on their − log 10 p value with a cut-off at p < 0.05 (Fig. 7F). Interestingly, all these processes were down-regulated as the genes related to these processes were significantly down-regulated in day 28 infected samples.

We also used the pathway profile scores from nCounter to construct a heat map and compared the pathway modulation between day 7 and day 28 infected samples. A total of 23 pathways were compared which were already pre-selected in the Alzheimer’s disease Nanostring Panel (Fig. 7G). Most of the pathways were over-expressed in day 28 infected mice in comparison to day 7 infected mice. These trends also corroborated with the findings of the REACTOME database where most of the processes, especially ubiquitin-mediated protein degradation, were down-regulated reflecting the over-activation of “unfolded protein response” in day 28 infected mice pathway profile score.

In summary, these findings suggest that *C. pneumoniae* infection leads to a differential regulation of Alzheimer’s disease genes with long term infection (day 28) down-regulating most of the genes related to folding of proteins and aiding in misaggregation.

## Discussion

In the current study, we showed that (1) *C. pneumoniae* rapidly infected both the olfactory and trigeminal nerves in mice, (2) *C. pneumoniae* entered the CNS via nerves within 24–72 h after intranasal inoculation and without concurrent blood infection, (3) injury to the nasal epithelium exacerbated peripheral nerve infection, but reduced brain infection, (4) *C. pneumoniae* inclusions in the olfactory nerve and bulb were associated with accumulations of Aβ, (5) the glial cells populating the olfactory/trigeminal nerves and brain supported *C. pneumoniae* replication, and (6) *C. pneumoniae* infection leads to differential regulation of Alzheimer’s disease related genes. Thus, *C. pneumoniae* can very rapidly spread from the periphery to the CNS via the nerves extending between the nasal cavity and the brain, without blood infection. To our knowledge, this study is the first report of Aβ deposition in response to *C. pneumoniae* infection of the primary olfactory nervous system, and the first time such rapid (72 h) deposition of Aβ in response to any bacterium in wild-type animals in vivo has been demonstrated.

The time-frame for infection of the CNS by *C. pneumoniae* was considerably faster than what has previously been shown (1 week–3 months^16–18^), which may be due to differences in the inoculation dose since we used a higher inoculation dose than two previous studies^16,50^ but lower than another^18^. Nevertheless, the time-frame is comparable to CNS invasion via cranial nerves by *Burkholderia pseudomallei*^32,43,44,52^, *Streptococcus pneumoniae*^53^, *Neisseria meningitidis*^54^, *Listeria monocytogenes*^55^ and now recently another *Chlamydia* species, *C. muridarum*^24^. The amoeba *Naegleria fowleri*^56^, as well as herpes simplex type virus type 1 (HSV-1)^57^, severe acute respiratory syndrome coronavirus 2 (SARS-CoV-2)^58–60^ and other coronaviridae^61^ can also invade the CNS via these two paths (shown in humans and/or animals).

Within the olfactory bulb, *C. pneumoniae* inclusions were detected in OECs within the nerve fibre layer/glomerular layer. Another bacteria, *Burkholderia pseudomallei* also accumulated within the nerve fibre layer/glomerular layer after intranasal inoculation, suggesting that the glia limitans acts to restrict further progression of bacteria into the deeper regions. However, with *C. pneumoniae* while we could easily detect the inclusion bodies, the much smaller infectious elementary bodies would likely be missed in our analyses of the tissue sections; thus it is possible that elementary bodies were present deeper in the olfactory bulb. As inclusion bodies were detected in the olfactory piriform cortex, it suggests that *C. pneumoniae* did progress deeper into the olfactory bulb as previously reported^15,16,18,50^.

Injury to the nasal epithelium has been shown to increase infection of the olfactory nerve and bulb by *B. pseudomallei*^27^ and to allow the entry of *S. aureus*, which does not normally invade cranial nerves, to enter the olfactory bulb^28^. We therefore hypothesized that epithelial injury may lead to increased *C. pneumoniae* invasion of the olfactory/trigeminal nerves, olfactory bulb and remaining parts of the brain. We found that epithelial injury resulted in increased *C. pneumoniae* load in the olfactory mucosa (which contains the fascicles of the olfactory nerve), olfactory bulb and trigeminal nerve. In contrast, injury did not alter *C. pneumoniae* invasion of the brain after 7 days. We have previously observed a similar result for *B. pseudomallei* in some mice, in which the nasal infection in itself caused massive peripheral infection and destruction of the nasal epithelium (more pronounced than in our epithelial injury model used in the current study). In these mice, *B. pseudomallei* invasion of the CNS was negligible^20^. We then hypothesized that this may be because glia in the olfactory nerve and outer layers of the bulb responded to both the injury and bacteria, secreting large amounts pro-inflammatory factors which limited CNS infection; this may also be the case for *C. pneumoniae* infection in the current study.

The ability to infect glia is considered key for CNS invasion via the cranial nerve paths^20,27,28,34,35^. We here showed that *C. pneumoniae* could infect, survive in and replicate (form inclusions) within glia from the PNS (OECs and TgSCs) and the CNS (astrocytes and microglia). This is the first-time infection of OECs and TgSCs (or other Schwann cells) by *C. pneumoniae* has been reported, however, we have recently shown that *C. muridarum* can infect OECs and TgSCs^24^. Whilst *C. pneumoniae* infection of cultured primary astrocytes and microglia has not been described, infection of astrocyte and microglial cell lines has been demonstrated^62–66^. Most relevantly, however, *C. pneumoniae* antigens have been detected inside both astrocytes and microglia in post-mortem human brains^9,11,67,68^. OECs, Schwann cells and astrocytes are all innate immune cells which can respond to and phagocytose bacteria, and microglia (the macrophages of the CNS) are well characterized professional phagocytes^31,69,70^. The fact that *C. pneumoniae* can form inclusions in these cells suggest that the bacteria, at least to some extent, can overcome phagocytic destruction; this may be one important mechanism by which this bacterium can invade and establish long-term infection of the CNS.

We also detected localized deposition of Aβ adjacent to *C. pneumoniae* IBs and in the olfactory bulb after 7 days and 28 days post inoculation. Diffuse/scattered Aβ immunoreactivity was also present in these tissues of control mice, however, the co-localisation of Aβ deposits and *C. pneumoniae* inclusions in inoculated mice was clear and distinct. Previous studies have demonstrated Aβ deposits near *C. pneumoniae*-infected areas of the cerebral cortex 1–4 months post intranasal inoculation^16^. One study reported that whilst there were not necessarily more Aβ deposits in the cortex of *C. pneumoniae*-infected animals, Aβ deposits in infected animals were morphologically different from those in control animals^18^. A previous long-term study showed that *C. pneumoniae* infection of the cerebral cortex preceded the peak of Aβ deposition^17^. In combination with the findings of the current study, it appears that Aβ secretion occurs in response to the infection. One reason may be that Aβ is secreted as an antimicrobial agent^12^ but alternatively it may be secreted in response to infection because of pathway activation for the processing of the APP protein into Aβ which is then secreted; future work can clarify the secretion and role of Aβ in this context.

The secretion of Aβ may thus be a normal immune response to any microbe that may invade the nervous system, and if infection clears, the deposited Aβ can be cleared by phagocytic glia^71^. It is, however, possible that if bacteria are not cleared and instead become persistent or latent in neural cells, continued Aβ deposition may occur, contributing to late-onset dementia and/or accelerating Aβ deposition in familial Alzheimer’s disease^7^. In the case of *C. pneumoniae*, one study in wild-type mice demonstrated that Aβ deposits resulting from infection were subsequently cleared^17^, whilst another study showed that the deposits did not disappear over several months^16^.

It is interesting that we observed Aβ deposits in the olfactory nerve earlier than in the bulb, as one study in an Alzheimer’s disease mouse model (APP/PS1 mice) showed that the terminal end of the olfactory nerve within the nasal olfactory epithelium is the first nervous system area to exhibit Aβ deposition, which then progresses to the olfactory bulb and other CNS areas^72^. As the mice in that study were kept in a standard animal holding facility (not specific pathogen free), perhaps exposure to infectious agents may have contributed to this early, peripheral deposition of Aβ (which likely would be much more pronounced in an Alzheimer’s disease model than in wild-type mice).

*Chlamydia pneumoniae* infection also resulted in up-regulation of key pathways involved in Alzheimer’s disease pathogenesis. The pathologic features of Alzheimer’s disease like activated microglia, production of inflammatory mediators and reactive oxygen species (ROS) were highly regulated in infected brain tissue at 28 days post inoculation as compared to 7 days post inoculation. Theses neuroinflammatory responses are considered a major driving factor in patients with neurodegeneration and Alzheimer’s disease pathology, which starts early in the course of the disease, prior to the formation of Aβ plaques in the brain^73^. Previous studies have shown that microglia and astrocytes act as host cells of *C. pneumoniae* in Alzheimer’s disease brain^9^. It has been shown that following infection, activated microglia and astrocytes secrete pro-inflammatory cytokines, including IL-1β, TNFα and IL-6 which are neurotoxic and may directly increase Aβ production via activation of β-secretase (BACE)^66,74^. BACE cleaves amyloid precursor protein and initiates the amyloid cascade. Microglia activation reduces the accumulation of Aβ in the brain by increasing its phagocytosis, clearance, and degradation^75^. However, the neuroinflammation associated with Alzheimer’s disease could be a double-edged sword because persistent microglia activation stimulated by the binding of microglia to Aβ can increase the production of inflammatory mediators and reactive oxygen species (ROS), which further amplifies the neuroinflammatory response causing chronic inflammation and neurodegeneration^76^.

Disturbance of endoplasmic reticulum (ER) function is emerging as a relevant factor driving neurodegeneration in Alzheimer’s disease^77^. Several reports have described manifestations of ER stress in post-mortem brain samples from Alzheimer’s disease patients^78^. Protein folding in the endoplasmic reticulum (ER) is an essential cell function and to safeguard protein production and ensure quality control, ER-stress triggers the activation of several biochemical pathways collectively referred to as the unfolded protein response (UPR). *Chlamydia* infection can induce cellular stress that impacts protein folding, thus inducing UPR activation however it is also proposed to modulate the UPR to promote their survival and replication^79^. Interestingly, we found UPR pathway being up-regulated in infected cortical tissues at 28 days post inoculation as compared to 7 days post inoculation*.* Intracellular pathogens like *Chlamydia* would benefit from UPR since increase in folding capacity and activation of lipid biosynthesis can sustain bacterial replication. However, if the ER stress due to infection is sustained and misfolded protein cannot be refolded or degraded, the cells can also directly increase Aβ production and associated neuroinflammation^80^. Conversely, Aβ oligomers have also been proposed to cause ER dysfunction leading to UPR mediated neurotoxicity and neuronal cell death^77^. We have also observed similar trends in our study where molecular pathways related to cell death like autophagy and apoptosis were up-regulated in cortical tissues at 28 days post inoculation.

In addition to considering key pathways, it is also useful to consider changes in individual gene expression. Long term *C. pneumoniae* infection (day 28) triggered down-regulation of most other key genes involved in AD pathogenesis. Most importantly there was downregulation of key protective heat shock protein (*Hspa1b or Hsp70-2*), associated with increased oxidative stress and initiation of AD pathology^81^. In addition, *Bag2,* a Bcl-2 associated co-chaperone gene which controls Hsp70 functionality was also downregulated leading to further failure of the system to protect cells from oxidative damage^82^. The long term infection also depressed the 26S proteasome ubiquitination system by downregulation of *Psmd8*^83^ and *Psmc6*^84^ leading to persistence of stress-induced protein aggregates. At a sub-cellular level, infection led to mitochondrial dysfunction evident by downregulation of *Ndufa5* (a structural subunit of complex I)^85^ and *Atp5j2*^86^. Nevertheless, all these gene modulations led to increased unfolded protein response, oxidative stress, and had higher disease association as evident by the biological processes heat map. In fact, the long-term infection was also associated with low expression of *Cd2ap* which has been previously associated with AD pathology aggravated by increased deposition of Aβ and Tau-induced neurotoxicity^87^.

In contrast to the downregulated genes, long-term infection was also associated with some repairing mechanisms, limiting the spread of further neuroinflammation. It led to higher expression of *Cdk18* which is a cyclin-dependent kinase and usually functions to clear DNA damages^88^. Thus, mechanisms inducing chromatin modification, transcription and splicing were highly reduced in them. Amongst the heat shock proteins, we found *Hspa4* to be upregulated which ensured that it maintained the disaggregating property of any misfolded proteins, thereby, preventing further damage and inducing tissue integrity^89^. Additionally, it also helped in sulfatase gene (*Sumf2*) maintenance which is associated with modulation of tissue homeostasis^90^. Interestingly, long term infection also induced expressions of *Ezr*^91^ and *Cldn5*^92^ which are associated with maintenance of actin cytoskeletal structure, synapse and tight junctions, respectively. This ensured that long-term infection induced greater organisation of neuronal cytoskeleton and/or dendritic structure while also maintaining synaptic transmission and reuptake. Additionally, further protection was provided by increased *Axl* activity, a phagocytotic gene (autophagy) important in maintaining homeostatic levels of major AD related lipoprotein, ApoE^93^. Long term infection also induced *Mbp* gene, which maintained myelination and prevented further neuroinflammation^94^. In contrast, upregulation of *Flt1*, a key gene in vascular endothelial growth factor (VEGF) regulation was a key feature in long term infection which could reflect cognition impairment due to higher Aβ and Tau deposition in AD etiopathogenesis, as observed in a previous study^95^. As a result of increased growth factor signalling, angiogenesis was induced with increased trophic activity demonstrating ongoing inflammatory activity.

## Conclusion

We have demonstrated the rapid invasion of the olfactory and trigeminal nerves, the olfactory bulb and brain (beyond the bulb) by *C. pneumoniae* in mice. In the olfactory nerve and bulb, *C. pneumoniae* inclusions were associated with localized deposits of Aβ, which appeared in the olfactory nerve earlier than in the bulb. We also showed that injury to the nasal epithelium led to increased bacterial load in the peripheral nerves and olfactory bulb, but did not increase load in the rest of the brain during the time course of our study. At the cellular level, we showed that *C. pneumoniae* can infect and replicate to form inclusion bodies within primary glial cells of the olfactory/trigeminal nerves and CNS. Of particular importance, *C. pneumoniae* infection led to the dysregulation of key pathways involved in Alzheimer’s disease pathogenesis up to 28 days after intranasal inoculation. From these results, we conclude that the nerves extending between the nasal cavity and the brain constitute invasion paths by which *C. pneumoniae* can rapidly invade the CNS and trigger genetic and molecular changes in the longer term which may contribute to the onset of Alzheimer’s disease pathogenesis.

## Supplementary Information

Supplementary Information.
