# Supplementary material for: Chlamydia pneumoniae can infect the central nervous system via the olfactory and trigeminal nerves and contributes to Alzheimer’s disease risk
Source: Sci Rep. 2022 Feb 17;12:2759. doi: 10.1038/s41598-022-06749-9 (PMC8854390; doi:10.1038/s41598-022-06749-9)
Supplement: Supplementary file 1 — Supplementary Information. [file 41598_2022_6749_MOESM1_ESM.docx]

**Supplementary information**

***Chlamydia pneumoniae* can infect the central nervous system via the olfactory and trigeminal nerves and contributes to Alzheimer’s disease risk**

Chacko A^1*^, Delbaz A^1*^, Walkden H^1^, Basu S^1^, Armitage CW^2^, Eindorf T^1^, Trim LK^3^, Miller E^1^, West NP^1^, St John JA^1,4#^, Beagley KW^3#^, Ekberg JA^1,4#,*^


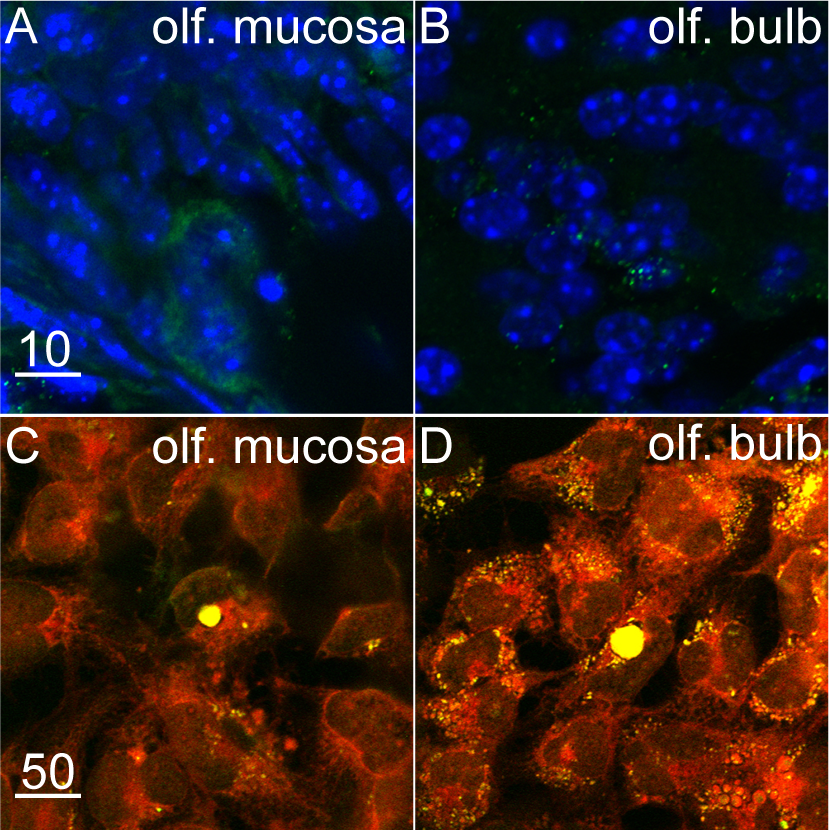


**Supplementary Figure 1.** *C. pneumoniae* invades the olfactory mucosa and olfactory bulb within 24 h after intranasal inoculation. Panels A-B show representative confocal microscopy z-stack images of the olfactory mucosa (A) and olfactory bulb (B) from inoculated mice. *C. pneumoniae* inclusions are shown in green (immunolabelling) with nuclei/DNA in blue (DAPI stain). Panels C-D show HE-p2 cells (red) 72 h after exposure to tissue homogenates from olfactory mucosa and olfactory bulb, with *C. pneumoniae* inclusion forming units (green, but appears yellow due to merge with red). Scale bars in µm.


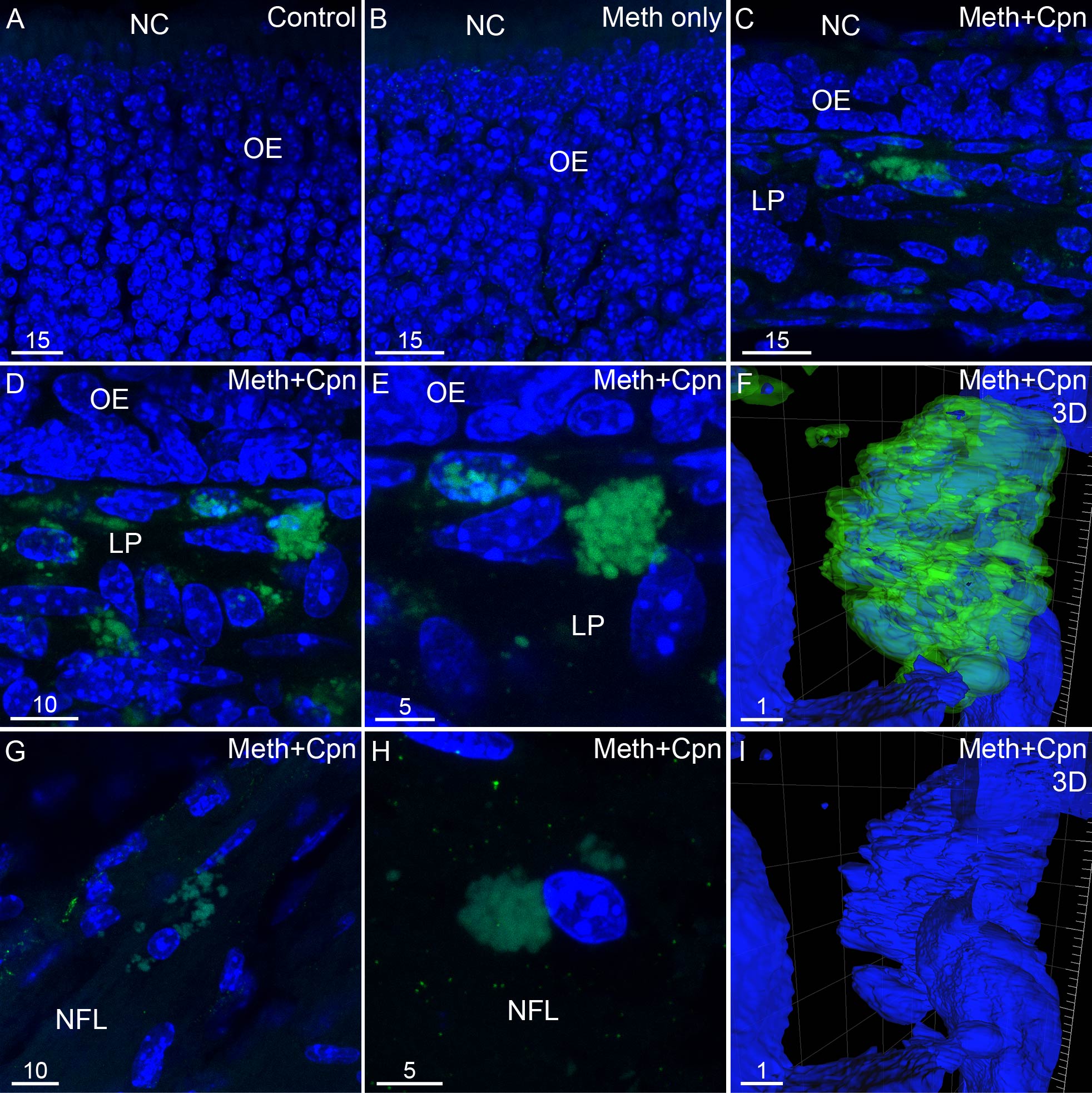


**Supplementary Figure 2.** Methimazole-induced epithelial injury, followed by intranasal inoculation with *C. pneumoniae*, causes infection of the olfactory nerve and bulb within only 24 hours of intranasal inoculation. Panels show representative images of tissue sections from both control (vehicle only; N = 3) and inoculated mice (*C. pneumoniae*; N = 3 for both three and seven days post intranasal inoculation). *C. pneumoniae* inclusions are shown in green (immunolabelling) with nuclei/DNA in blue (DAPI stain). Panels show maximum projection of confocal microscopy z-stacks. (A-B) Images show the olfactory epithelium (OE), near the nasal cavity (NC), in control mice (A) and mice treated with methimazole (Meth; B) but not inoculated with *C. pneumo*niae. (C-H) Images show tissue sectioned from mice treated with methimazole followed by intranasal inoculation with *C. pneumoniae* (Meth+Cpn). (C) C. pneumoniae (green) in the lamina propria (LP) beneath the OE. (D-E) C. pneumoniae inclusions (green) in the LP; panel E shows a higher magnification view of panel D. (F) A three-dimensional reconstruction of the *C. pneumoniae* inclusion shown in panel E. (I) Same three-dimensional reconstruction as F but showing only the DAPI staining (blue). Bacterial DNA within the inclusion body, distinct from host cell DNA, can be detected. (G-H) C. pneumoniae inclusions (green) within the nerve fibre layer (NFL) of the olfactory bulb. Scale bars in µm.
